# Editorial: Methods in diagnosing heart failure

**DOI:** 10.3389/fcvm.2024.1365006

**Published:** 2024-01-23

**Authors:** Giulia Elena Mandoli, Laura Spaccaterra, Erberto Carluccio, Riccardo Maria Inciardi

**Affiliations:** ^1^ Department of Medical Biotechnologies, Division of Cardiology, University of Siena, Siena, Italy; ^2^ Cardiology and Cardiovascular Pathophysiology, S. Maria Della Misericordia Hospital, University of Perugia, Perugia, Italy; ^3^ Department of Medical and Surgical Specialties, Radiological Sciences and Public Health, Institute of Cardiology, University of Brescia, Brescia, Italy

**Keywords:** heart failure, ejection fraction, echocardiography, advanced imaging, multimodality imaging

**Editorial on the Research Topic** Methods in diagnosing heart failure

## Introduction

Heart failure (HF) is a clinical syndrome that has a complex pathophysiology. It is related to a functional or structural cardiac abnormality that results in inadequate cardiac output at rest and/or during exercise and/or during elevated intracardiac pressures. It can present with a range of symptoms and signs which should be discriminated. Frequently associated with multiple comorbidities, it results in many hospitalizations and high morbidity and mortality. Due to these reasons, HF has an important economic impact on healthcare systems.

This Research Topic presents an update on some parameters useful for a better characterization of patients with HF.

## The role of left ventricle ejection fraction: HF classification

Based on the left ventricular ejection fraction (LVEF), HF can be classified in:

- Heart failure with reduced ejection fraction (HFrEF), when the LVEF is below 40%;
- Heart failure with mildly reduced ejection fraction (HFmrEF), when the LVEF is between 41% and 49%;
- Heart failure with preserved ejection fraction (HFpEF), when the LVEF is >50% in a patient with symptoms and signs of HF and/or evidence of structural or functional cardiac abnormalities and/or raised natriuretic peptides.

Although this is the definition, it should be considered that the LVEF is a continuous variable that can change during the natural history of patients with HF but also according to loading condition. The latest European Association of Cardiology guidelines (1), in fact, recommend assessing the trajectory of the LVEF.

Ding et al. investigated the frequency and prognostic implications of changes in the LVEF trajectory. A total of 2,429 patients were enrolled, among which 46% had HFpEF, 37% HFrEF, and 17% HFmrEF. At a median follow-up of 3.78 years, 20% of the patients showed an increase in the LVEF, 65% had a static LVEF, and 15% of the patients had a worsening LVEF.

The results showed that patients with a worsening LVEF over time had an increased risk of events, including HF-related hospital admissions and all-cause mortality; on the contrary, patients with an intensified LVEF showed a significantly reduced risk of composite endpoints.

## Role of right ventricle evaluation in HFrEF

Chronic HFrEF is characterized by the heart's inability to efficiently pump blood throughout the body but is also associated with a variable increase in the left heart filling pressures, leading to a remodeling in pulmonary vasculature up to biventricular failure.

A comprehensive assessment of the right chambers is paramount to apply a correct risk stratification of HFrEF patients and, as a result, a better therapeutic allocation.

The study presented by Benes et al. suggests that, in patients with HFrEF, it should be important not only to evaluate traditional parameters for RV function assessment, such as TAPSE and fractional area change (FAC), but also advanced echocardiographic measures such as right ventricular (RV) strain for the evaluation of longitudinal deformation of the right ventricle. On the contrary, the authors underline the importance of integrating the mentioned parameters, which are focused on the properties of RV myocardium, with the RV size as well. In fact, in patients with HFrEF, RV dilatation should not be viewed as a final stage of RV disease but as an independent parameter of RV dysfunction, which can carry a prognostic value. Evaluation of both RV size and degree of dysfunction in one score, called the RV global dysfunction score, can thus reflect more accurately the degree of RV disease. In a population of 836 stable HFrEF patients, subjects with a lower degree of RV dysfunction but a larger RV size had similar outcomes to those with worse RV dysfunction but smaller RV size.

## New methods for HFrEF evaluation

One of the goals of HF management is to intercept as soon as possible clinical or subclinical indicators of increasing congestion to prevent acute decompensation and the need for hospitalization, modifying the medical therapy as soon as possible.

A possible role can be played by ballistocardiography (BCG) and seismocardiography (SCG), techniques that can assess the inotropic state by measuring the body movements induced by cardiac contraction and blood flow in the cardiac chambers and major vessels. In the KINO-HF study by De Keyzer et al., 30 patients with HF and an impaired LVEF were matched with 30 patients with an LVEF >50%. Sixty seconds registration of Kinocardiography (KCG), a combination of BCG and SCG, was recorded after cardiac ultrasound acquisition, and the kinetic energy was computed in different phases of the cardiac cycle as a marker of cardiac mechanical function. The results showed that KCG can distinguish HF patients with impaired systolic function from a control group, showing that this can be a method for screening patient with abnormal LVEF.

## HFpEF

Nearly half of the population with HF presents a preserved EF, with a similar outcome but less therapeutic options. The number of patients affected is estimated to increase as the society ages and body mass indexes increase. Recently, some diagnostic algorithms have been proposed to help diagnose HFpEF. In the study by Kim et al., the authors evaluated the relative importance of left atrial strain (LAS) regarding the *Heart Failure Association Pre-test assessment; Echocardiography and natriuretic peptide, Functional testing, Final etiology* (HFA-PEFF) score (2) in predicting a score of 5 or 6. In particular, the study shows how left atrial reservoir strain (LASr), in more than 2,700 patients was associated with a high HFA-PEFF score, independent from the E/e' ratio or LV relative wall thickness, and that this parameter was a strong predictor of diastolic function compared to existing parameters used to assess it.

In conclusion, this Research Topic included various diagnostic points that can be useful for a better and earlier evaluation of patients with HF.
